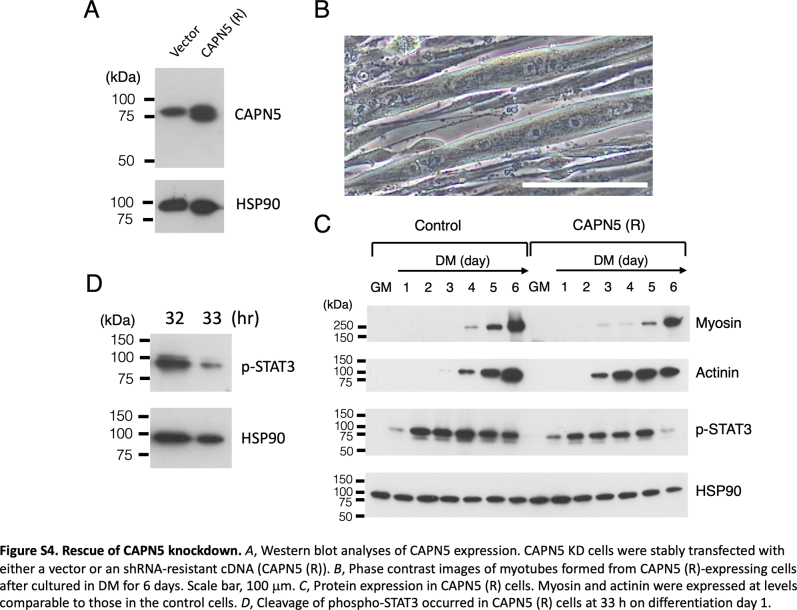# Calpain-5 regulates muscle-specific protein expression and nuclear positioning during myoblast differentiation

**DOI:** 10.1016/j.jbc.2024.107842

**Published:** 2024-09-30

**Authors:** Nobuhiro Morishima, Yoshihiro Ito

**Affiliations:** 1 Nano Medical Engineering Laboratory, Cluster for Pioneering Research, RIKEN, Wako, Japan; 2 Emergent Bioengineering Materials Research Team, Center for Emergent Matter Science, RIKEN, Wako, Japan

**Keywords:** calcium, calpain, Myc (c-Myc), myogenesis, myonuclei, proteolysis, skeletal muscle, STAT3

## Abstract

Intracellular calcium dynamics is key to regulating various physiological events. Myotube formation by myoblast fusion is controlled by the release of Ca^2+^ from the endoplasmic reticulum (ER), and the calpain (CAPN) family is postulated to be an executioner of the process. However, the activation of a specific member of the family or its physiological substrates is unclear. In this study, we explore the involvement of a CAPN in myoblast differentiation. Time-course experiments showed that the reduction in potential substrates of calpains, c-Myc and STAT3 (signal transducer and activator of transcription 3) and generation of STAT3 fragments occurred multiple times at an early stage of myoblast differentiation. Inhibition of the ER Ca^2+^ release suppressed these phenomena, suggesting that the reduction was dependent on the cleavage by a CAPN. CAPN5 knockdown suppressed the reduction. *In vitro* reconstitution assay showed Ca^2+^- and CAPN5-dependent degradation of c-Myc and STAT3. These results suggest the activation of CAPN5 in differentiating myoblasts. Fusion of the *Capn5* knockdown myoblast efficiently occurred; however, the upregulation of muscle-specific proteins (myosin and actinin) was suppressed. Myofibrils were poorly formed in the fused cells with a bulge where nuclei formed a cluster, suggesting that the myonuclear positioning was abnormal. STAT3 was hyperactivated in those fused cells, possibly inhibiting the upregulation of muscle-specific proteins necessary for the maturation of myotubes. These results suggest that the CAPN5 activity is essential in myoblast differentiation.

We had previously demonstrated that Ca^2+^ release from the endoplasmic reticulum (ER) *via* ER Ca^2+^ channels in differentiating myoblasts is necessary for myoblasts to form a multinuclear syncytium—myotube—by cell fusion (1, 2, 3). When ER Ca^2+^ is released into the cytosol, ER stress occurs because ER Ca^2+^ depletion interferes with the correct folding of *de novo* synthesized proteins in the ER (4). Primarily, ER stress activates cytoprotective signaling pathways (unfolded protein response, UPR) (5, 6). Blocking the UPR in myoblasts inhibits muscle-specific protein synthesis and cell fusion (1). Therefore, ER stress signaling in differentiating myoblasts may play a defensive role and act as a driving force for differentiation.

In addition to ER stress, ER Ca^2+^ depletion activates store-operated Ca^2+^ entry (7). To replenish Ca^2+^ in the ER, the ER Ca^2+^ sensor protein stromal interaction molecule 1 (STIM1) oligomerizes and activates the Ca^2+^ channels at the plasma membrane (8), which induces Ca^2+^ influx from the extracellular space into the cytosol. Therefore, ER Ca^2+^ depletion in differentiating myoblasts causes a change in Ca^2+^ dynamics in the cytosol due to Ca^2+^ release from the ER and Ca^2+^ uptake from the extracellular space. These changes in Ca^2+^ dynamics in differentiating myoblasts prompted us to explore additional roles of Ca^2+^ during myoblast differentiation other than the activation of the UPR. Therefore, we investigated proteolysis using Ca^2+^-dependent proteases, such as calpains (9). The calpain (CAPN) family is a group of cysteine proteases (CAPN1–16 in humans and mice) whose activation is Ca^2+^-dependent. Gene mutations of calpains in humans and mice lead to pathological phenotypes, suggesting their physiological significance (10). Hence, calpains are executioners of Ca^2+^-dependent processes in various physiological events. However, the physiological function of calpains, the physiological substrates, and their mechanism of activation *in vivo* are poorly understood (10).

Previous studies have suggested that calpains are involved in regulating myoblast differentiation. Chemical inhibitors broadly effective against multiple calpains significantly suppress myoblast fusion in rats (11) and chickens (12). However, to our knowledge, proteolysis mediated by calpains in differentiating myoblasts has not been reported. An example of CAPN-mediated proteolysis during myoblast differentiation is the cleavage of c-Myc, presumably by calpain-1 or calpain-2 in murine myotubes formed after myoblast fusion (13). The N-terminal fragment of c-Myc (Myc-nick) generated by cleavage at Lys298 likely protects cells from apoptosis (14). Considering the potential yet poorly explored role of calpains in myoblast differentiation and the largely unknown substrates of calpains, we aimed to shed light on the role of calpain-mediated proteolysis in myoblast differentiation. In this study, we investigated the cleavage of candidate calpain substrates in the mouse myoblast cell line—C2C12—during ER Ca^2+^ depletion. We previously detected oligomer formation of STIM1 in C2C12 cells on differentiation day 1 (3). In our experiment, C2C12 cells exited the cell cycle by day 2 and subsequently started cell fusion, which was detected under a microscope on day 3 or later (1). Therefore, to determine the involvement of a CAPN in myogenesis, we examined whether calpain proteolysis occurred in differentiating myoblasts on day 1.

## Results

### Cleavages of transcription factors in differentiating myoblasts during ER Ca^2+^ depletion

**Figure 1:**
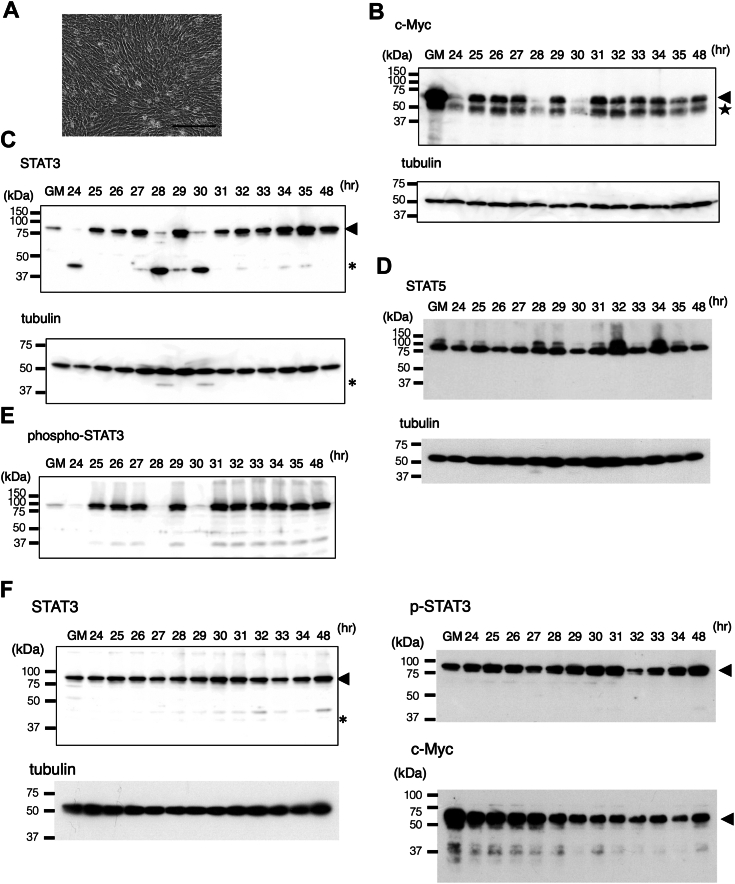
**Transient reduction of c-Myc and STAT-3 in C2C12 cells on differentiation day 1.***A*, morphology of C2C12 cells on differentiation day 1. C2C12 cells grown to a sub-confluent state followed by treatment with DM (24 h). A phase-contrast image of cells in a confluent state is shown. Bar, 100 μm. *B*, Western blot analysis of c-Myc in differentiating myoblast. Cells were recovered from the culture plates at 1 h intervals (24–35 h) and 48 h for Western blot analysis. After probing with an anti-c-Myc antibody, the blot was reprobed with anti-α-tubulin to check a loading control. The *arrowhead* and *star* indicate c-Myc (60 kDa) and probable isoforms of c-Myc (52 kDa), respectively. GM, proliferating cells in growth medium (GM). *C*, Western blot analysis of STAT3. The *arrowhead* indicates STAT3 (88 kDa), and an *asterisk* indicates the cleavage product (42 kDa). Cleavage products of α-tubulin (43 kDa) are also indicated by an *asterisk*. *D*, Western blot analysis of STAT5. *E*, the blot used in *B* was reprobed with an anti-phospho-STAT3 antibody. *F*, inhibition of Ca^2+^ release from the ER suppresses transient reductions of STAT3, phospho-STAT3, and c-Myc. Western blot analysis of STAT3, phospho-STAT3, and c-Myc. The *asterisk* indicates the position at which p42 would be detected if STAT3 cleavage occurred. The blot was reprobed with anti-α-tubulin to detect the loading control.

To detect a CAPN activation in differentiating myoblasts, we examined c-Myc and STAT3 because these transcription factors were cleaved in healthy cells: murine myotubes (13) and activated human platelets (15). C2C12 cells cultured in differentiation medium (DM) became confluent within 24 h (Fig. 1*A*), and the cell morphology did not change during differentiation day 1 (24−35 h). Western blot analysis showed that transient reduction of c-Myc (approximately 60 kDa) occurred at 24, 28, and 30 h (Fig. 1*B*). However, neither Myc-nick (42 kDa) (13) nor other truncated c-Myc proteins were detected. Analyzing the same cell lysate samples, we found that STAT3 (88 kDa) almost disappeared at 24, 28, and 30 h, and a cleavage product of approximately 42 kDa (p42) appeared simultaneously (Fig. 1*C*). Based on the position of the epitope (within amino acid residues 1–100 at the N-terminus), STAT3 underwent limited proteolysis in the middle region of the STAT3 which differs from the cleavage site near its C-terminus in activated platelets (15). The cleavage of STAT3 in platelets were accompanied by STAT5 cleavage, which are reportedly cleaved by calpain-1 (15). However, STAT5 cleavage was not detected in differentiating myoblasts (Fig. 1*D*). We occasionally detected α-tubulin fragments of approximately 43 kDa at the time points of STAT3 cleavage (Fig. 1*C*), albeit at a cleavage efficiency much lower than that of STAT3. The size of the cleavage product was different from the 50 kDa fragments generated by calpain-1 or calpain-2 cleavage in the brain under pathological conditions (16, 17). These results suggest the activation of a protease is different from that responsible for cleavage in myotubes (13), activated platelets (15), or the brain (16, 17).

**Fig. S1:**
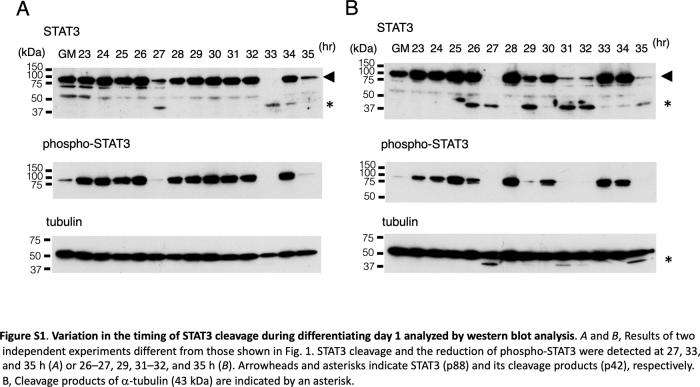


STAT3 is activated *via* phosphorylation and dimerization (18). Phospho-STAT3 (Tyr705) (88 kDa) in differentiating myoblasts almost disappeared at 24, 28, and 30 h (Fig. 1*E*). The reduction in the level of phospho-STAT3 was comparable to or greater than that of STAT3 (Fig. S1, *A* and *B*). Phospho-STAT3 may be more efficiently cleaved by the protease than the unmodified form, and its cleavage is a sensitive marker of the activation of the presumed protease. We observed that the timing or frequency of the protease activation varied at 27, 33, and 35 h in one experiment (Fig. S1*A*); at 26 to 27, 29, 31 to 32; and at 35 h in other experiments (Fig. S1*B*). The fluctuation may depend on batches of cells and/or the different cell culture conditions, such as the cell density during myoblast transfer into DM.

To examine the dependence of STAT3 cleavage on ER Ca^2+^ depletion, we inhibited ER Ca^2+^ channels in differentiating myoblasts (3) by the combined use of an IP_3_R inhibitor (19) and a ryanodine receptor inhibitor (20). Both inhibitors efficiently suppressed the cleavage of STAT3 (Fig. 1*F*). Similarly, phospho-STAT3 was reduced by 66% (an average from two technical replicates) at 32 h but did not completely disappear (Fig. 1*F*). The reduction in c-Myc was not apparent (Fig. 1*F*). These results support the idea that the assumed protease in differentiating myoblasts depends on the change in the Ca^2+^ dynamics.

### CAPN5 is responsible for the transient cleavages

**Fig. S2:**
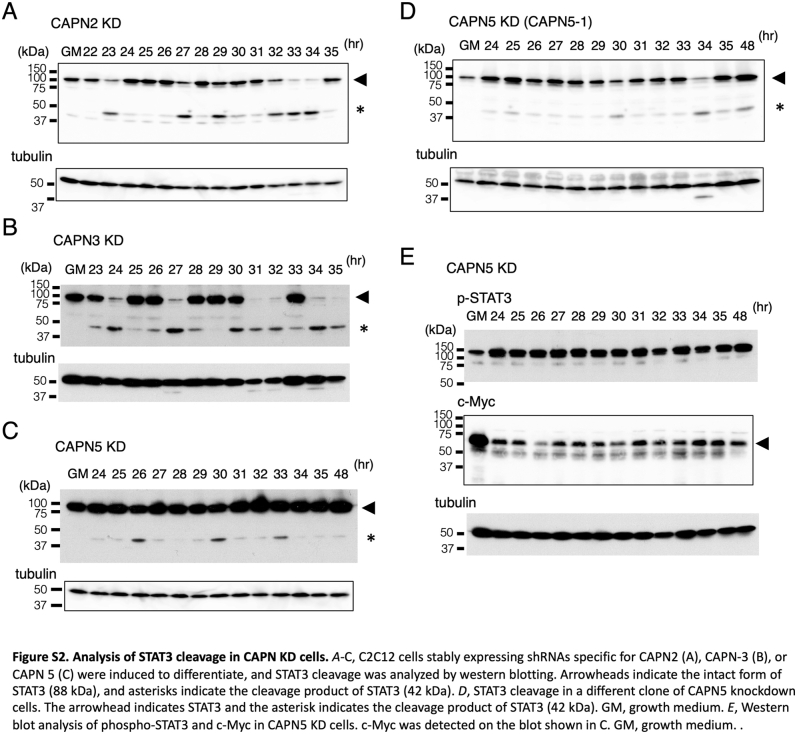


**Fig. S3:**
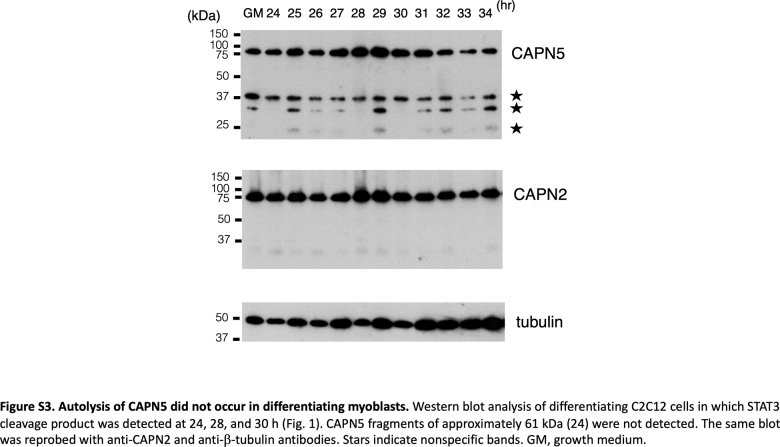


We knocked down CAPN2, -3, or -5 expressions in C2C12 cells by stably overexpressing specific small hairpin RNAs (shRNAs). CAPN2 and CAPN5 are constitutively expressed in most cells *in vivo*, whereas CAPN3 is specific to skeletal muscle cells (21). In CAPN2 and CAPN3 stable knockdown (KD) cells, STAT3 cleavage occurred efficiently (Fig. S2, *A* and *B*). However, in *CAPN5* KD cells, the percentages of cleavage products (p42) to the total amount (p42 + p88) were 15% at 26 h, 15% at 30 h, and 8% at 33 h (Fig. S2*C*), contrasting to those in the control cells that were up to nearly 100%. In an independent clone of CAPN5 KD cells that expressed a different shRNA for gene knockdown (CAPN5-1), mild cleavage of STAT3 was observed at 25, 30, and 34 h with cleavage ratios of 5, 26, and 48%, respectively (Fig. S2*D*). In addition, neither phospho-STAT3 nor c-Myc disappeared, with a slight decrease in c-Myc (25, 30, and 34 h) (Fig. S2*E*). These results suggest that CAPN5 is responsible for the cleavage of (phospho-)STAT3 and the reduction of c-Myc. Calpains can be autoprocessed after exposure to calcium (reviewed in (22)), although the functional significance of the autoproteolysis of CAPN remains controversial. CAPN5 (and CAPN2) processing was not detected in the control cells on differentiation day 1 (Fig. S3).

**Figure 2:**
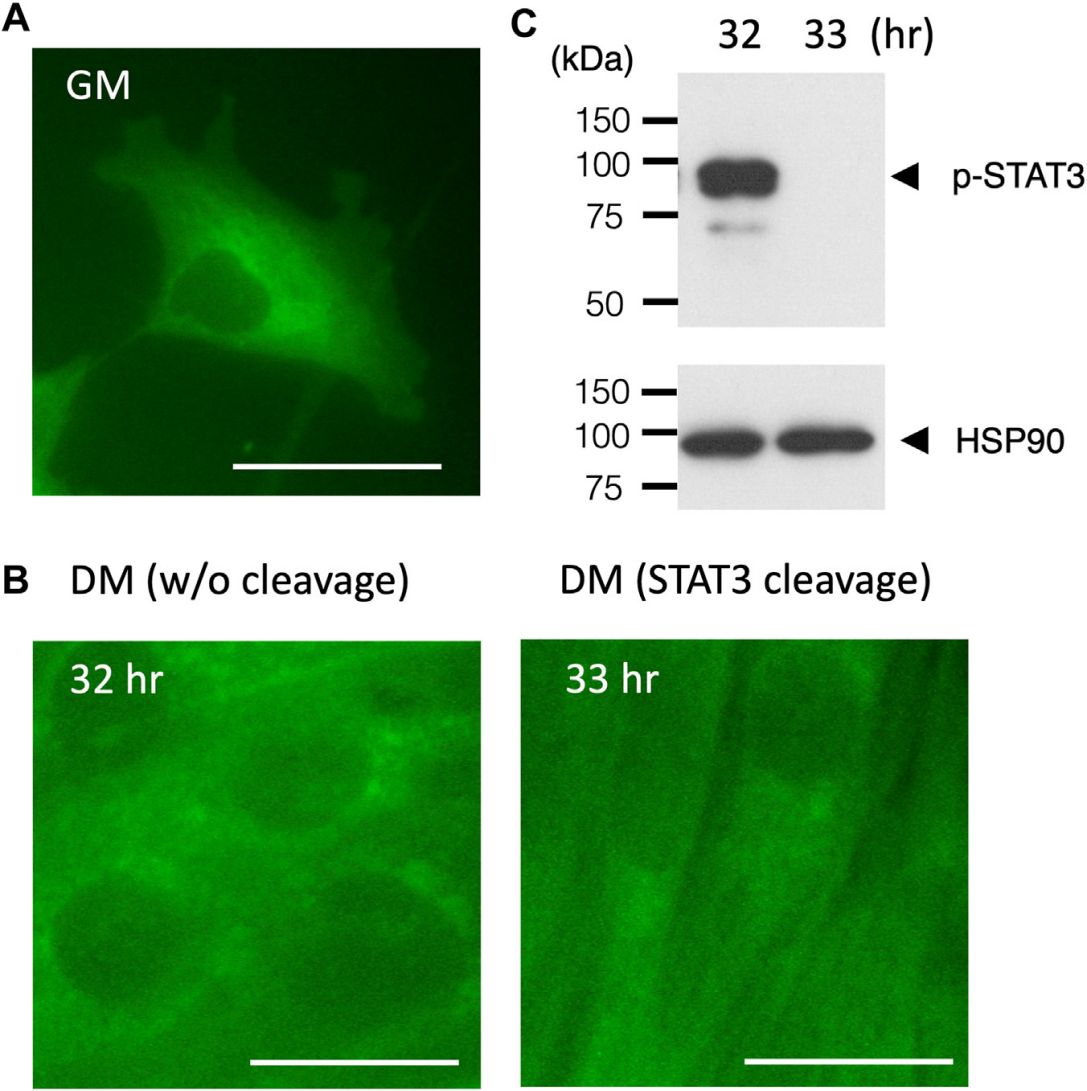
**Cytoplasmic localization of CAPN5.***A*, CAPN5 was detected by overexpression of mAG1-tagged proteins in C2C12 cells. A representative image of cells in GM. Bar, 50 μm. *B*, intracellular localization of CAPN5 in C2C12 cells in DM (32 and 33 h). Bars, 50 μm. *C*, Western blot analysis of phospho-STAT3 (p-STAT3). Phospho-STAT3 (88 kDa, *arrowhead*) was cleaved at 33 h. The blot was reprobed with anti-HSP90 antibody to check a loading control.

To investigate the intracellular localization of CAPN5, we observed CAPN5 tagged with a GFP-related monomeric fluorescent protein (23) in differentiating myoblasts. The majority of a C-terminally tagged CAPN5 was detected in the cytosol of proliferating myoblasts (Fig. 2*A*), and the change in the localization pattern was not detected throughout day 1 with or without phospho-STAT3 cleavage (Fig. 2, *B* and *C*). Possibly, neither the preferential localization of CAPN5 in the nucleus nor its extensive translocation to the nucleus was the mechanism by which phospho-STAT3 was more efficiently cleaved by CAPN5 than unmodified STAT3 (see Discussion).

### *In vitro* cleavage of potential substrates by CAPN5

**Figure 3:**
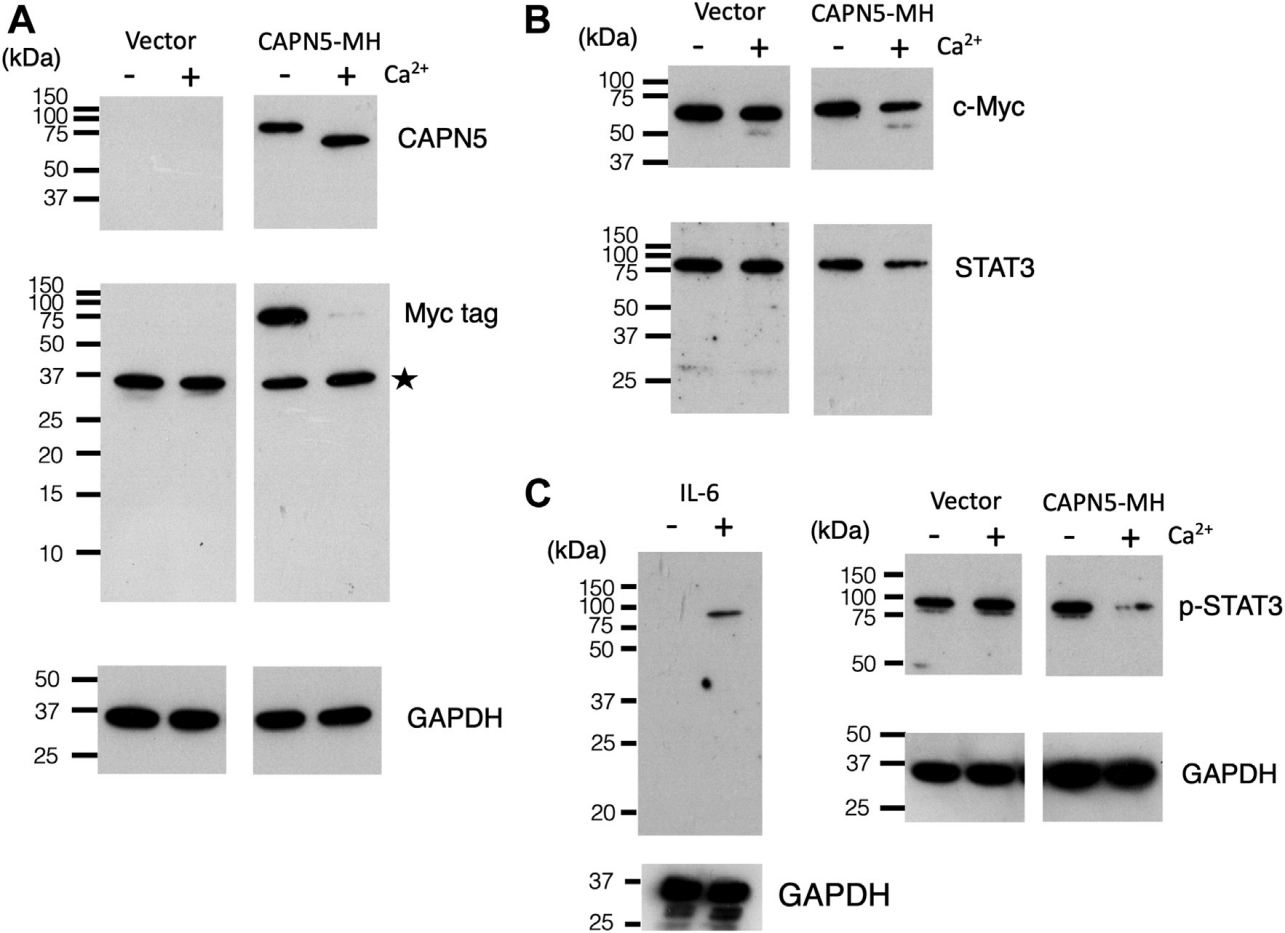
***In vitro* cleavage of candidate substrate of CAPN5.** MCF-7 cells were transiently transfected with a vector or the CAPN5-MH (Myc-His tag) cDNAs. Crude cell lysates prepared from the transfectants were treated with (+) or without (−) 2 mM CaCl_2_. Representative data from two transfection experiments are shown. Signal intensities of c-Myc, STAT3, and phospho-STAT3 (p-STAT3) were normalized to GAPDH. *A*, Western blot analyses of CAPN5 and the Myc tag. The Myc tag antibody detected a nonspecific protein of approximately 37 kDa (a *star*). GAPDH was used as a loading control. *B*, the blot shown in *A* was reprobed with anti-c-Myc or anti-STAT3 antibodies. *C*, (*left*), phospho-STAT3 in MCF-7 cells treated with (+) or without (−) 50 ng/ml human IL-6 for an hour. (*right*), cell lysates after the Ca^2+^ treatment were separated.

To examine whether CAPN5 cleaves c-Myc and STAT3 *in vitro*, we produced recombinant murine CAPN5 proteins with a C-terminal Myc-His (MH) tag in the human breast cancer cell line MCF-7. Crude lysates of the MCF-7 transfectants prepared in 1 mM EDTA were treated with 2 mM CaCl_2_ *in vitro*. Furthermore, we investigated the possibility of CAPN5 autoprocessing. Western blot analysis showed that CAPN5-MH (76 kDa) was efficiently converted into a smaller species (approximately 62 kDa) after incubation with Ca^2+^ (Fig. 3*A*). An anti-Myc tag antibody did not detect CAPN5-MH after Ca^2+^ treatment (Fig. 3*A*). These results suggest that CAPN5-MH was cleaved by autoprocessing at a site near or within the C-terminal tag, in addition to another site that generated the 62 kDa fragments. Moreover, Ca^2+^-dependent conversion of a C-terminally tagged CAPN5 has been observed in SH-SY5Y cell lysates (24). Murine CAPN5 proteins without the C-terminal tag were not autoprocessed in lysates treated with Ca^2+^ (data not shown). CAPN5 has a C-terminal C2 domain, which is a Ca^2+^-dependent membrane-binding module (25). The presence of the MH tag at the C-terminus of CAPN5 may have affected membrane binding (26), the three-dimensional structure of the CAPN5 molecule, or both, facilitating the autoproteolysis of CAPN5-MH.

C-Myc in crude lysates of MCF-7 cells was digested by CAPN5-MH. The intact form of c-Myc was reduced to 57% (averages from two biological replicates) after incubation of the CAPN5-MH lysates in the presence of CaCl_2_ (Fig. 3*B*). In the control lysates, c-Myc levels decreased to 91% after incubation possibly due to digestion by endogenous enzymes. Additionally, c-Myc was reduced to 69% in the CAPN5 (without the tag) lysates with Ca^2+^ (data not shown), suggesting that the CAPN5 processing is not strictly required for activation. These results indicated that CAPN5 cleaves c-Myc.

After treatment with CaCl_2_, STAT3 was reduced to 64% (averaged from two biological replicates) in the crude lysate of CAPN5-MH overexpressing cell, while the level of STAT3 did not change in the control lysates (Fig. 3*B*). The p42 fragment of STAT3, detected in differentiating myoblasts was absent in the crude lysates, possibly due to its instability in MCF-7 cell lysates (Fig. 3*B*). We induced STAT3 phosphorylation in MCF-7 cells by treating them with IL-6 (Fig. 3*C*), activating the JAK/STAT pathway (27) before preparing cell lysates. In CAPN5-MH lysates, Phospho-STAT3 was reduced to 68% (an average from two biological replicates) after Ca^2+^ treatment. However, the digestion of phosphorylated STAT3 was not detected in the control lysates (Fig. 3*C*). These results support the idea that the proteolytic activity of CAPN5 is responsible for the reduction of c-Myc and (phospho)-STAT3 in differentiating myoblasts.

### CAPN5 knockdown in myotubes caused hyperactivation of STAT3 and insufficient formation of myofibrils

**Figure 4:**
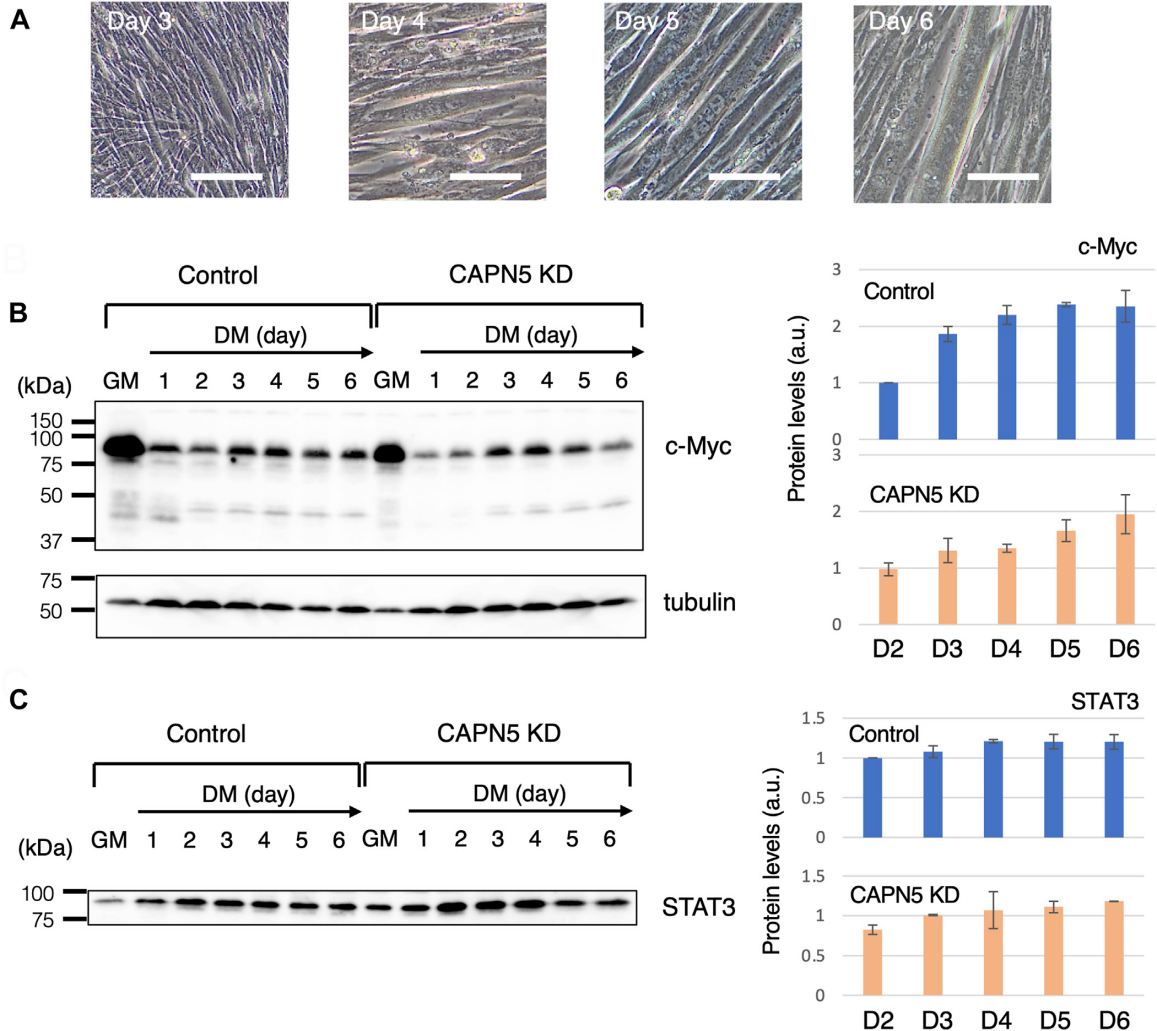
**Comparison of the protein levels between the control and CAPN5 knockdown cells from differentiation days 2 to 6.***A*, representative images of myotubes formed by fusion of control cells. Bars, 100 μm. Western blotting for c-Myc (*B*) and STAT3 (*C*). The same blot was repeatedly used for immunostaining. α-tubulin was used as a loading control. GM, growth medium; DM, differentiation medium. The *bar graphs* shows the protein levels of c-Myc (*B*) and STAT3 (*C*) determined from the data of two biological replicates. The protein levels were normalized to α-tubulin and are presented in the *bar graphs* as the ratio to the value in the control cells on day 2. a.u., arbitrary unit.

**Figure 5:**
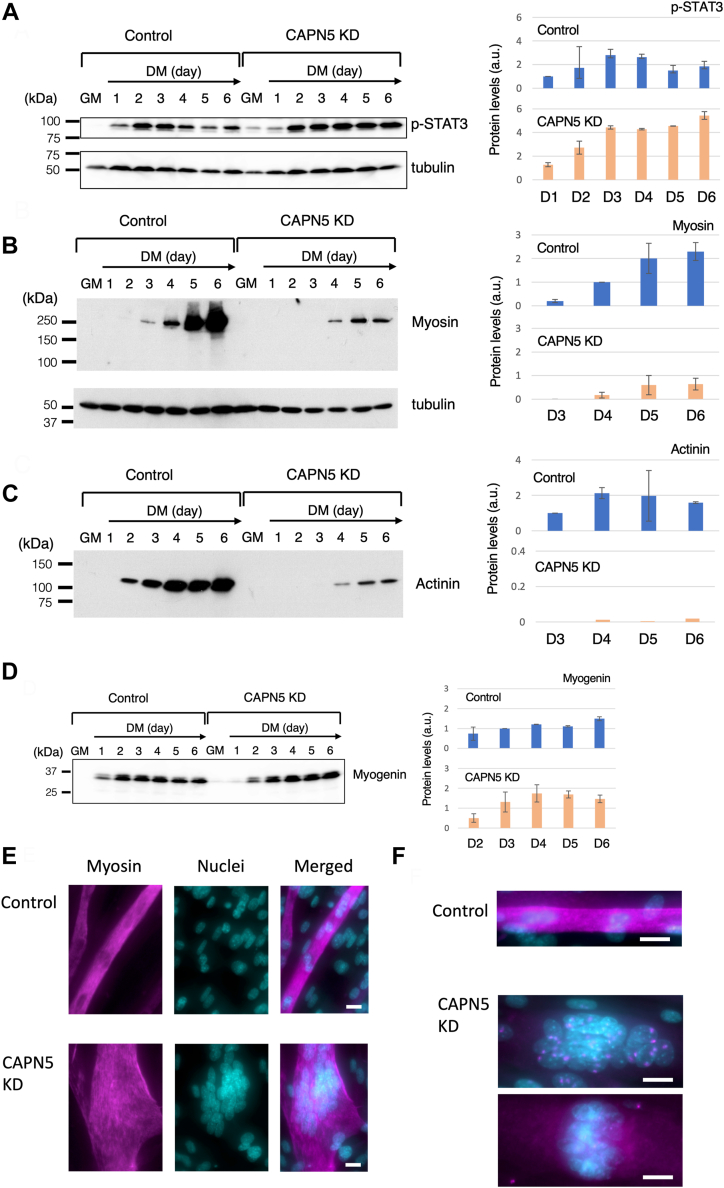
**Abnormalities of the CAPN5 knockdown cells in protein expression and cell morphology.***A*–*D*, (*left*), representative data of Western blot analyses are shown. (*right*), The *bar graphs* show the protein levels of phospho-STAT3 (*A*), myosin (*B*), actinin (*C*), and myogenin (*D*) determined from the data of two biological replicates. The protein levels were normalized to α-tubulin or HSP90 and are presented in the *graphs* as the ratio to the value in the control cells on day 1 (*A*), day 4 (*B*), or day 3 (*C* and *D*). a.u., arbitrary unit. The data of α-tubulin in (*A*) is a replicate of that shown in Figure 4*B*. *E*, myosin was immunostained in myotubes formed from the control cells or the CAPN5 KD cells cultured in DM for 6 days. Bound antibodies were visualized by biotin-conjugated anti-mouse IgG antibody and streptavidin-conjugated Alexa Fluor 488. Nuclei in the myotubes were labeled with Hoechst 33342. Myosin and nuclei are shown in a *magenta* and a *blue* pseudo-color, respectively. (*upper*), control cells; (*lower*), CAPN5 KD cells. Scale bars, 20 μm. *F*, actinin was immunostained in myotubes cultured in DM for 6 days. (*top*), control cells; (*middle* and *bottom*), CAPN5 KD cells. Actinin was detected as speckled structures on the nuclei (*middle*) or it was faintly immunostained in CAPN5 KD cells (*bottom*). Scale bars, 20 μm.

To examine the effects of suppressing CAPN5 cleavage, we compared the levels of the CAPN5 substrates between control cells and CAPN5 KD cells on Day 2 or later, during which cell fusion occurred (Fig. 4*A*). Western blot analyses indicated that the levels of c-Myc and STAT3 in both control and CAPN5 KD cells remained relatively constant from Day 3 (c-Myc) or Day 2 (STAT3) to Day 6, and were comparable between the two groups (Fig. 4, *B* and *C*). However, the patterns of variation in phospho-STAT3 levels differed from each other. In control cells, phospho-STAT3 increased after day 1, peaking on Days 3 and 4 before decreasing to 60 to 70% on Days 5 and 6 (Fig. 5*A*). This peak likely reflects the requirement for active STAT3 during *in vitro* myoblast differentiation (28). In contrast, phospho-STAT3 also increased in CAPN5 KD cells on Days 2 and 3, but these cells maintained higher phospho-STAT3 levels up to Day 5 and further increased on day 6. Phospho-STAT3 levels in CAPN5 KD cells were approximately 1.5-fold higher on Days 3 and 4, as well as more than twice on Days 5 and 6 compared to those in the control cells (Fig. 5*A*). These results suggest that the reduction in CAPN5 activity augmented STAT3 phosphorylation during the later stages of myoblast differentiation.

Next, we examined the levels of major components of myofibrils, myosin, and actinin. In control cells, myosin was detected by Western blot analysis on day 3, with levels increasing daily. However, myosin in CAPN5 KD cells was not detected until day 4, and its levels of myosin were approximately one-fourth of those in the control cells on Days 5 and 6 (Fig. 5*B*). Actinin exhibited an even more pronounced deficiency in induction. It was detected on day 2 and onward in the control cells. However, actinin in CAPN5 KD cells was approximately 1% of those in the control cells on days 5 and 6 (Fig. 5*C*). These results suggest that CAPN5 is crucial for the proper induction of muscle-specific proteins.

We investigated myogenin, a member of the MyoD family because it plays a central role in the induction of muscle-specific proteins (29). Myogenin is upregulated after the induction of differentiation in DM. Western blot analysis (Fig. 5*D*) showed that myogenin, represented by the 33 to 35 kDa doublet band (corresponding to unphosphorylated and phosphorylated myogenin), was induced in the control cells on day 1, and remained almost constant from day 2 onward. In CAPN5 KD cells, myogenin levels were approximately half of those in the control cells on Days 1 and 2. However, on days 3 onward, the myogenin levels were comparable to those in the control cells. These results suggest that myogenin expression was not deficient in CAPN5 KD cells; nevertheless, muscle-specific proteins were poorly induced.

Immunostaining showed that myosin formed continuous structures along the long axis of a myotube made of control cells (Fig. 5*E*). In contrast, myosin in fused cells derived from CAPN5 KD cells appeared as fragments or aggregates, some measuring several micrometers in length or width (Fig. 5*E*, myosin fluorescence image). Additionally, the fluorescent images indicated abnormal morphology of fused cells and nuclear crowding (details to be described below). The CAPN5 KD cells exhibited poor expression of actinin, as confirmed by weak immunostaining. Actinin appeared as speckled stains on the nuclei in most (>90%) of the fused cells formed from CAPN5 KD cells. Other cells displayed weak staining of actinin throughout the cytosol (Fig. 5*F*). These results suggest that the formation of myofibrils in CAPN5 KD cells was incomplete, possibly because of the low expression of myosin and actinin.

### CAPN5 KD inhibited the progress of myonuclear positioning in myotubes

**Figure 6:**
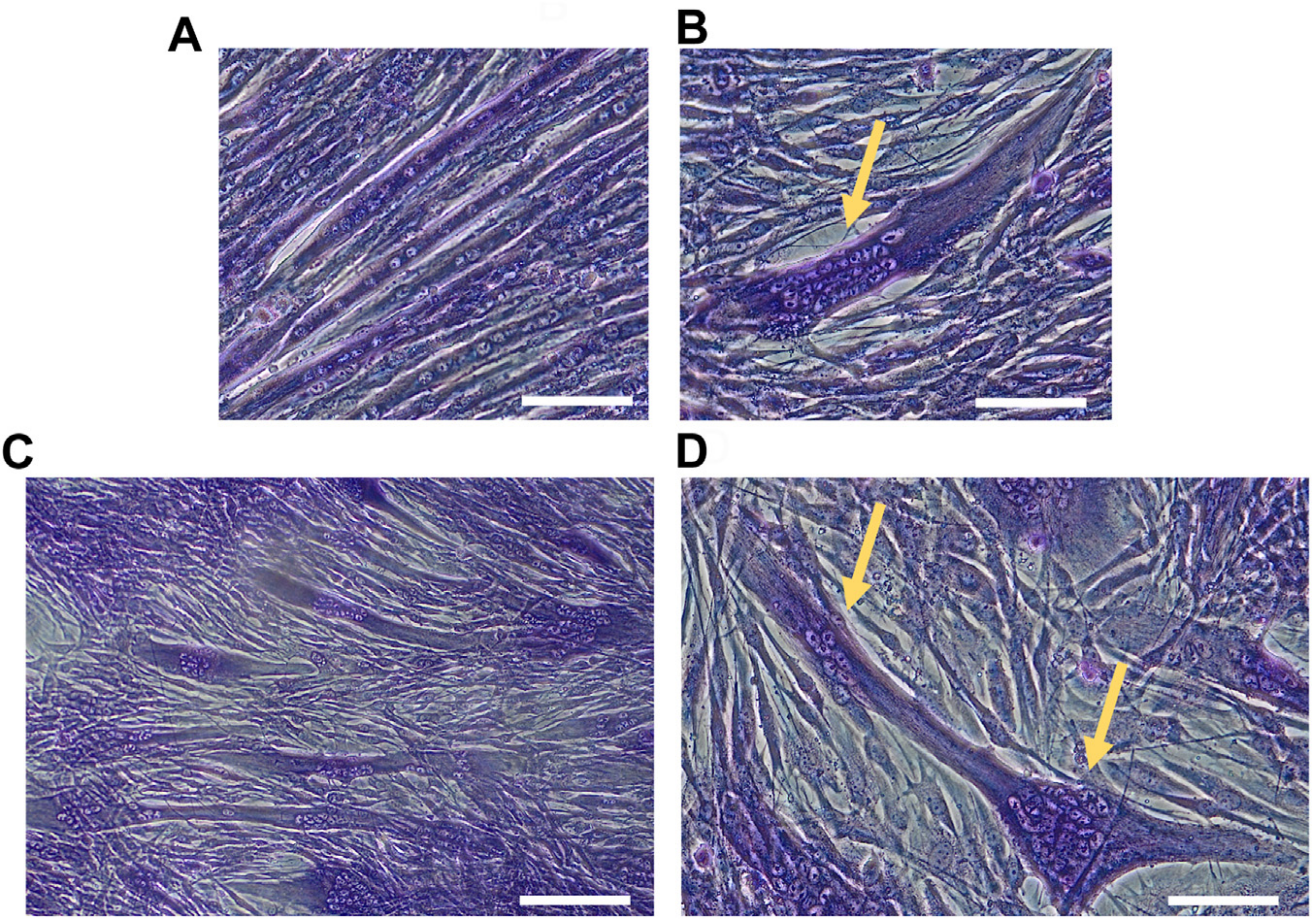
**Nuclei in the myotubes of the CAPN5 knockdown cells formed clusters.***A–D*, May-Grünwald-Giemsa staining of myotubes on differentiation day 6. Myotubes of the control cells (*A*) or the CAPN5 KD cells (*B*–*D*). *Arrows* indicate the clusters of nuclei. Scale bars: 100 μm (*A*, *B*, and *D*) or 200 μm (*C*).

May-Grünwald-Giemsa staining of nuclei (30) revealed unusual nuclear positioning in CAPN5 KD cells. Unlike the parental cells (Fig. 6*A*), the myonuclei in CAPN5 KD did not disperse along the axis of entire cells. Alternatively, it formed a cluster of a few tens of nuclei (Fig. 6*B*). The cells were enlarged around the cluster. Such clusters were present in nearly all multinuclear cells formed from CAPN5 KD cells (Fig. 6*C*). In relatively large fused cells, more than two clusters of nuclei were detected within a single cell (Fig. 6*D*). Myonuclear clusters were also observed in other CAPN5 KD cells (CAPN5-1) (data not shown). According to a recent model of myonuclear positioning during myotube formation (31), nuclei in a mononuclear myoblast rapidly migrate toward the center of the syncytium shortly after cell fusion to form a cluster of nuclei; however, they spread apart as myotubes mature. Our findings suggest that a substantial decrease in CAPN5 activity inhibits the maturation of myotubes and causes a defect in the dispersion of the myonuclear cluster.

To examine if the phenotype of CAPN5 knockdown was rescued, we overexpressed a variant of the CAPN5 cDNA resistant to the shRNA. The increase in CAPN5 expression (approximately 1.7 times (Fig. S4*A*)) resulted in the restoration of normal nuclear positioning (Fig. S4*B*) in myotubes, along with significant induction of myosin and actinin (Fig. S4*C*). Additionally, hyperphosphorylation of STAT3 did not occur at the later stages of differentiation (Fig. S4*C*), and cleavage of phospho-STAT3 was observed during differentiation on day 1 (Fig. 4*D*). These results confirmed that the phenotype of CAPN5 KD cells was dependent on the considerable decrease of CAPN5 in C2C12 cells.

### Effects of excessive STAT3 activation

**Figure 7:**
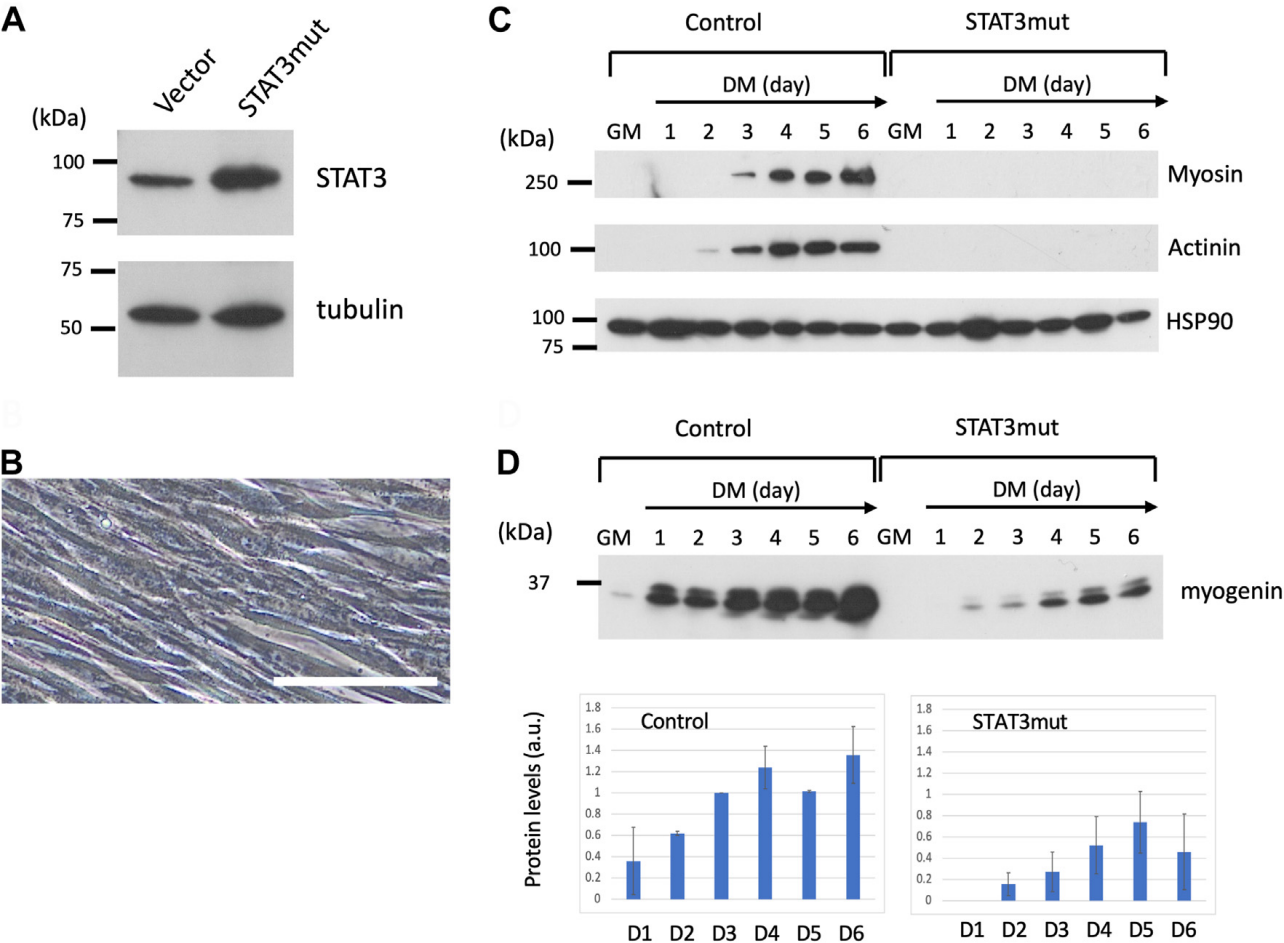
**Effects of constitutively active STAT3.***A*, Western blot analyses of STAT3 expression. C2C12 cells were stably transfected with either a vector or constitutively active STAT3 cDNA (STAT3mut). *B*, phase contrast images of STAT3mut cells cultured in DM for 6 days. Scale bar, 100 μm. *C*, myosin and actinin induction was absent from STAT3mut cells cultured in DM. *D*, (*upper*), Western blot analyses of myogenin. Representative data are shown. The same blot was repeatedly used for immunostaining with anti-HSP90 antibody and is shown in (*C*). (*lower*), the bar graph shows the protein levels of myogenin determined from the data of two biological replicates.

To examine the effects of STAT3 hyperactivation, we overexpressed STAT3 mutant proteins (STAT3mut) that were spontaneously dimerized (32) at a level approximately 0.7-fold of the endogenous STAT3 (Fig. 7*A*). The transfectants did not fuse in the DM (Fig. 7*B*). Myosin and actinin were not induced even on Day 6 (Fig. 7*C*). These results suggest that hyperactivation of STAT3 inhibits the induction of these muscle-specific proteins. The induction of myogenin started no earlier than day 2. The expression levels were approximately 30% of those in the control cells up to Day 6 (Fig. 7*D*). Moreover, complete suppression of cell fusion in the STAT3mut-overexpressing cells may be due to either lower levels of myogenin or hyperactivation of STAT3 from the beginning of differentiation induction in DM, or both. The inhibitory effects of STAT3 on myogenesis have been suggested (33, 34).

## Discussion

This study suggests that CAPN5 was activated for approximately 60 min during each activation in differentiating myoblasts. The intact forms of the cleavable proteins were restored through *de novo* synthesis shortly after transient cleavage. This pattern of proteolysis contrasts with that observed under pathological conditions, where calpains (presumably calpain-1 or calpain-2) remain activated for hours until cell death occurs. This difference may be attributed to the enzymatic characteristics of CAPN5, which requires a relatively high concentration of calcium (35). Such a high Ca^2+^ concentration is likely achieved relatively quickly and within a limited space in the cellular microenvironment (36). Activation may occur in a minority of CAPN5 in the nucleus because c-Myc and phospho-STAT3 are good substrates of CAPN5. A not mutually exclusive mechanism of the transient activation is CAPN5 deactivation by other CAPNs. Interspecies cleavage has been proposed as a mechanism of calpain inactivation (37). Intracellular calcium plays a central role in regulating myoblast differentiation (reviewed in (38)), and proteins that respond to changes in calcium concentration, such as calcineurin, whose activity peaks in myoblasts before 24 h in DM (39), have been identified as regulators. It should be investigated how these proteins, including calpains, cooperate in response to the changes in calcium dynamics in differentiating myoblasts.

Although the CAPN5 activation in differentiating myoblasts is transient, the activation timing may be critical. Differentiation Day 1 corresponds to the transition stage of myoblasts from cell cycle exit to terminal differentiation. If the cleavages of c-Myc and phospho-STAT3 result in their inactivation, the transcriptome is affected, and the proteome may change accordingly.

As the cleavage of α-tubulin suggests, CAPN5 probably has other substrates in differentiating myoblasts. A search for the physiological substrates of CAPN5 is ongoing. Recently, mutations in the *Capn5* gene were identified as the cause of neovascular inflammatory vitreoretinopathy (40). Identifying CAPN5 substrates would contribute to the molecular analysis of the pathological condition and the development of the diagnostics.

Skeletal muscles contain hundreds of myonuclei distributed evenly along the periphery of individual muscle cells. Such nuclear localization is assumed to be necessary to achieve proper transcription and translation levels in the entire myofiber area (41). The dispersed positioning of nuclei is achieved through ordered and well-regulated processes, including the myonuclear spread after myoblast fusion (31). Current hypotheses of myonuclear positioning emphasize the essential role of myofibrils or muscle-specific adaptor proteins that connect the nucleus and the noncentromeric-microtubule-organizing center (42, 43). The results of this study suggest the importance of myofibrils in the event. However, the synthesis of proteins other than myosin and actinin, which are induced upon myoblast differentiation may also be impaired by the reduction of the CAPN5 activity. Therefore, the essential functions of adaptor proteins in myonuclear positioning cannot be excluded.

The conventional understanding of the proteome change during myoblast differentiation and the establishment of the muscle-specific proteome is that these processes are principally driven by the action of myogenic transcription factors (44). This study suggests that CAPN5 provides another layer of the regulation of myogenesis. The possibility that CAPN5 provides another layer of regulation in myogenesis warrants further investigation into whether the cleavage products of CAPN5 substrates are functionally important. The calpain family produces functional fragments that modulate the activity of intact forms and other regulatory proteins (45).

## Experimental procedures

### Cell culture

C2C12 cells were purchased from RIKEN Cell Bank (Tsukuba, Ibaraki, Japan). Cells were grown in growth medium (GM) containing Dulbecco’s Modified Eagle Medium (Gibco 12430-062, Thermo Fisher Scientific) supplemented with 20% (v/v) fetal bovine serum (FBS) (Gibco 10099-141, Thermo Fisher Scientific), and 50 U/ml penicillin-streptomycin (Gibco 15070-063, Thermo Fisher Scientific). Cells were grown to sub-confluence for the differentiation studies on culture plates coated with 150 μg/ml of gelatin (G-2500, Sigma-Aldrich). Differentiation was induced in the DM containing 2% horse serum (Gibco 16050-130, Thermo Fisher Scientific) instead of 20% FBS and 1 μg/ml of insulin (I0516, Sigma-Aldrich). For time-course western blotting, proliferating C2C12 cells were divided equally into Corning 60 mm culture plates (430166, Corning, NY, USA) and grown in GM overnight until they reached a sub-confluent state the next day. For the inhibition study of the ER Ca^2+^ channels, 100 μM 2-aminoethoxydiphenyl borate (2-APB) and 20 μM dantrolene (D9754 and D9175, respectively, Sigma-Aldrich) were included in DM (3).

MCF-7 cells (Cell Resource Center for Biomedical Research, Tohoku University) were cultured in Invitrogen RPMI1640 medium (Thermo Fisher Scientific) supplemented with 10% FBS and 50 U/ml penicillin-streptomycin. MCF-7 cells were transiently transfected with *Capn5* cDNAs using FuGENE HD Transfection Reagent (Promega) according to the manufacturer’s protocol. After 28 h, the cells were recovered by scraping and stored at −80 °C until use. For phosphorylation of STAT3, transfectants were treated before the cell recovery for 1 h with 50 ng/ml of human IL-6 (PeproTech, Thermo Fisher Scientific) (46).

### shRNA plasmid

Synthetic primers used to construct shRNA plasmids are listed in Table 1. The pairs of synthetic DNAs for generating CAPN siRNA templates were annealed and cloned into the pGeneClip hMGFP vector (Promega) according to the manufacturer’s protocol. Plasmid DNA was amplified in *E. coli* DH5α (BioDynamics Laboratory) and purified using a NucleoSpin Plasmid QuickPure kit (Macherey-Nagel).

**Table 1:** Synthetic DNAs used for the construction of shRNA plasmids

Negative control
Sense, 5′-TCTCAATTCTCCGAACGTGTCACGTCTCGAGACGTGACACGTTCGGAGAATTCT-3′
Antisense, 5′-CTGCAGAATTCTCCGAACGTGTCACGTCTCGAGACGTGACACGTTCGGAGAATT-3′


CAPN2
Sense, 5′-TCTCGGAGCTGCTCTTTGTGCATTCCTTCCTGTCAGAATGCACAAAGAGCAGCTCCCT-3′
Antisense, 5′-CTGCAGGGAGCTGCTCTTTGTGCATTCTGACAGGAAGGAATGCACAAAGAGCAGCTCC-3′


CAPN3
Sense, 5′-TCTCGCAGTACCGTCTCAAGCTTCTCTTCCTGTCAAGAAGCTTGAGACGGTACTGCCT-3′
Antisense, 5′-CTGCAGGCAGTACCGTCTCAAGCTTCTTGACAGGAAGAGAAGCTTGAGACGGTACTGC-3′


CAPN5
Sense, 5′-TCTCGGTGGAAGAGAACCGCCAATACTTCCTGTCATATTGGCGGTTCTCTTCCACCCT-3′
Antisense, 5′-CTGCAGGGTGGAAGAGAACCGCCAATATGACAGGAAGTATTGGCGGTTCTCTTCCACC-3′


CAPN5 version 2 (CAPN5-1)
Sense, 5′-TCTCGCCGGTACTTTACTGACATCACTTCCTGTCATGATGTCAGTAAAGTACCGGCCT-3′
Antisense, 5′-CTGCAGGCCGGTACTTTACTGACATCATGACAGGAAGTGATGTCAGTAAAGTACCGGC-3′

BamHI/XhoI fragments containing the shRNA plasmid inserts were amplified by PCR using PrimeStar HS DNA polymerase (Takara Bio) and cloned into the XhoI/BamHI sites of the pHULK piggyBac C-terminal CometGFP vector (ATUM) using the In-Fusion HD Cloning Kit (Takara Bio). Primers used for amplification were complementary to partial sequences of the pGeneClip hMGFP vector and pHULK piggyBac C-terminal CometGFP (sense: 5′-TTGAAGAGCCGGATCC CTAAGGACCAGCTTCTTTG-3′; antisense, 5′-TGAATTCGGACTCGAG GTTAACATCGATGCGGC-3′).

### Gene knockdown by shRNA plasmids

C2C12 cells were transfected with shRNA plasmids using the TransIT-LTI transfection reagent (Mirus Bio) according to the manufacturer’s protocol. Stably transfected cells were selected for 12 days in GM containing 2 μg/ml puromycin (Sigma-Aldrich). The effect of gene knockdown in the transfectants was examined using western blotting. We identified the shRNA sequences that had reduced protein levels by at least 70% (data not shown). We examined the efficiency of gene knockdown by CAPN3-specific shRNAs by transient co-transfection of CAPN3 cDNA and shRNAs into MCF-7 cells, according to a previously described method (47). Briefly, MCF-7 cells grown in a 10 cm dish were transfected with *Capn3* cDNA (2.5 μg) and shRNA (2.5 μg) using SuperFect Transfection Reagent (Qiagen, Venlo). At 24 h post-transfection, cells were harvested, lysed, and analyzed using western blotting.

### Cloning and expression of the c-Myc, CAPN3, CAPN5, and STAT3 cDNAs

Primers used for the construction of c-Myc, CAPN3, CAPN5, and STAT3 plasmids are listed in Table 2. c-Myc cDNA was amplified from mouse liver cDNA pools (Zyagen) using PrimeStar HS DNA polymerase (Takara Bio). C-Myc cDNA was cloned into the NheI/BamHI sites of the Invitrogen pcDNA3.1 (−) vector (Thermo Fisher Scientific). The coding sequence of c-Myc cDNA was PCR-amplified and cloned into the NheI/BamHI sites of the pHULK piggyBac C-terminal CometGFP vector using the In-Fusion HD Cloning Kit. Primers used for the second amplification were complementary to the partial sequences of the pcDNA3.1 (−) vector and pHULK piggyBac C-terminal CometGFP (Table 2). For generating the Myc-nick plasmid, an antisense primer was used for the second amplification to truncate the c-Myc cDNA at the 298th codon followed by a stop codon (TAA). C2C12 transfectants stably expressing c-Myc or Myc-nick were established by transfection with the TransIT-LTI transfection reagent as described above.

**Table 2:** Primers used for the construction of c-Myc, CAPN3, and CAPN5 plasmids

Cloning of c-Myc
Sense, 5′-GTTTGAAGGCTGGATTTCCTTTGGGC-3′
Antisense, 5′-GATTCCAGCTCCTCCTCGAGTTAGGTCAGT-3′


Amplification of c-Myc cDNA
Sense, 5′-CATGGGAAGAGCTAGCCATGCCCCTCAACGTGAA-3′
Antisense, 5′-TTGAAGAGCCGGATCCTTATGCACCAGAGTTTCGAAGCT-3′


Amplification of Myc-nick cDNA
Sense, 5′-CATGGGAAGAGCTAGCCATGCCCCTCAACGTGAA-3′
Antisense, 5′-TTGAAGAGCCGGATCCTTACTTGAGGACCAGTGGGCT-3′


Cloning of *Capn3*
Sense, 5′-CACTATTTGAGCTGGTCAGAAGCCAGTCAG-3′
Antisense, 5′-GTGCTATGGACAACCCAGTACAAGCTAAGTAGACAG-3′


Amplification of *Capn3* cDNA
Sense, 5′-GGACTCAGATCTCGAGCCATGCCAACTGTTATTAGTCCAACTG-3′
Antisense, 5′-GGAGAGGGGCGGATCCTCAGGCATACATGGTAAGCTGCAGC-3′


Cloning of *Capn5*
Sense, 5′-GGACGTGGTCACCATGTTCTCCTG-3′
Antisense, 5′-GACATGCGAGTAGTGGTGAGGACTAGTGG-3′


Amplification of *Capn5* cDNA (for pcDNA3.1 (−) vector)
Sense, 5′-ACCCAAGCTGGCTAGCcATGTTCTCCTGCGCGAAGG-3′
Antisense, 5′-TACCGAGCTCGGATCcTCAGACAGCCGTGAGAGAGGCAC-3′


Amplification of *Capn5* cDNA (for pcDNA3.1 (−)/myc-His A vector)
Sense, 5′-ACCCAAGCTGGCTAGCcATGTTCTCCTGCGCGAAGG-3′
Antisense, 5′-TACCGAGCTCGGATCcTCAGACAGCCGTGAGAGAGGCAC-3′


Site-directed mutagenesis of *Capn5* cDNA
Sense, 5′-CTATAAGGTGGAAGAGAACAGGCAGTACCGTATGCACAGCCTAC-3′
Antisense, 5′-GTAGGCTGTGCATACGGTACTGCCTGTTCTCTTCCACCTTATAG-3′


Amplification of siRNA resistant *Capn5* cDNA (for PB-EF1α-MCS-IRES-Neo PiggyBac vector)
Sense, 5′-CTACTCTAGAGCTAGCCATGTTCTCCTGCGCGAAGGC-3′
Antisense, 5′-ATTCGAATTCGCTAGCTCAGACAGCCGTGAGAGAGGCAC-3′


Cloning of STAT3 cDNA
Sense, 5′-GACTGCAGCAGGATGGCTCAGT-3′
Antisense, 5′-CAGCTTCTGGTTTCAGCTCCTCACATG-3′


Amplification of STAT3 cDNA (for pcDNA3.1 (−) vector)
Sense, 5′-CTCGCTAGCcATGGCTCAGTGGAACCAGCTG-3′
Antisense, 5′-CTCGGATCCTCACATGGGGGAGGTAGCAC-3′


Amplification of STAT3 cDNA (for p3xFLAG-CMV-10 vector)
Sense, 5′-CTCGAATTCtATGGCTCAGTGGAACCAGCTGC-3′
Antisense, 5′-CTCGGATCCTCACATGGGGGAGGTAGCACAC-3′


Site-directed mutagenesis of STA3 cDNA
Sense, 5′-GGGCTATAAGATCATGGATTGTACCTGCATCCTGGTGTCTCCAC-3′
Antisense, 5′-GGGCTATAAGATCATGGATGCGACCAACATCCTGGTGTCTCCAC-3′


Amplification of STAT3mut cDNA (for pHULK piggyBac C-terminal CometGFP vector)
Sense, 5′-CATGGGAAGAGCTAGCCATGGACTACAAAGACCATGACGG-3′
Antisense, 5′-CGGATCCACCCTCGAGAATTCAACAGGCATCTACTGAGTGGACC-3′

The *Capn3* cDNA containing short 5′- and 3′ UTR sequences were amplified using PrimeStar HS DNA polymerase from mouse skeletal muscle cDNA pools (Zyagen). The coding sequence of *Capn3* cDNA was PCR-amplified and cloned into the XhoI/BamHI sites of the Clontech pIRES2-AcGFP1 vector (Takara Bio) using an In-Fusion HD Cloning Kit.

The *Capn5* cDNA was cloned using the same strategy used for *Capn3* cDNA cloning. First, the coding sequence with flanking UTR sequences was amplified from mouse liver cDNA pools (Zyagen). The coding sequences of the *Capn5* cDNA were amplified using two different sets of primers and were cloned into the NheI/BamHI sites of the pcDNA3.1 (−) and the NheI/BamHI sites of the pcDNA3.1 (−)/myc-His A (Thermo Fisher Scientific), respectively. The siRNA-resistant CAPN5 cDNA was constructed as follows. The coding sequence of *Capn5* cDNA was mutated at three sites within the region corresponding to the shRNA sequence using a QuikChange II site-directed mutagenesis kit (Agilent Technology). The mutated coding sequence was PCR-amplified and cloned into the NheI site of the PB-EF1α-MCS-IRES-Neo PiggyBac (System Biosciences). The cDNA of CAPN5 tagged with monomeric Azami Green (mAG1) was constructed by a multiple-insert cloning method. Monomeric Azami Green (mAG1) cDNA was kindly provided by Atsushi Miyawaki (RIKEN Center for Brain Science). The CAPN5 coding sequence in which the stop codon was removed and the mAG1 coding sequence were PCR-amplified and cloned into the NheI/XhoI sites of the pHULK piggyBac C-terminal CometGFP vector using an In-Fusion HD Cloning Kit, according to the manufacturer’s protocol (Takara Bio).

The STAT3 cDNA was amplified from mouse liver cDNA pools using two different primer sets and cloned into the XhoI/BamHI sites of the pcDNA3.1 (−) vector. The coding sequence of STAT3 was amplified and transferred to the EcoRI/BamHI sites of the p3xFLAG-CMV-10 vector (Sigma-Aldrich). The coding sequence of STAT3 cDNA was mutated at two sites (GCG to TGT, and AAC to TGC) corresponding to Ala662Cys and Asn664Cys mutations using a QuikChange II site-directed mutagenesis kit. The mutated coding sequence and the 3′ untranslated sequences that contained the polyA signal were PCR amplified and were cloned into the NheI/XhoI sites of the piggyBac C-terminal CometGFP vector using an In-Fusion HD Cloning Kit.

### Western blotting

The cells were lysed in a radioimmunoprecipitation assay buffer containing protease and phosphatase inhibitors (sc-24948, Santa Cruz Biotechnology). The protein concentration was quantified using a Protein Assay Dye Reagent Concentrate (5000006, Bio-Rad Laboratories), and BSA (Pierce 23209, Thermo Fisher Scientific) was used as the standard. The extracted proteins (10 μg, unless otherwise stated) were separated into 8%, 12%, or 14% SDS-PAGE gels. After electrophoresis, western blotting was performed on polyvinylidene difluoride membranes (Millipore Immobilon-P PVDF Membrane, Merck) using a Rapid Semi-Dry Blotter (WSE4045, ATTO) and EzBlot transfer buffer (AE1460, ATTO). Western blot analysis was performed as previously described (47) using 5% nonfat dry milk (31149-75, Nacalai Tesque) in Tris-buffered saline (pH 7.6) containing 0.1% Tween 20 (P7949, Sigma-Aldrich) as a blocking reagent. According to the manufacturer’s protocol, immunostained proteins were detected using the Amersham ECL Select Kit (RPN2235, Cytiva). Image analysis of the western blots was performed using either an ImageQuant LAS4000 mini imaging system (Cytiva) or Hyperfilm ECL (Cytiva) according to the manufacturer’s protocol. Chemiluminescent signals captured on the films were quantified using ImageJ (National Institutes of Health). Immunostained western blots were reprobed after stripping the bound antibodies with the EzReprobe stripping reagent (WSE-7240, ATTO) according to the manufacturer’s protocol.

### *In vitro* activation of CAPN5

MCF-7 cells were transiently transfected with murine *Capn5* cDNAs as described in the section Cell Culture (second paragraph) above. Cells were suspended in 50 mM Tris-HCl (pH 7.6) containing 150 mM NaCl, 1 mM EDTA, 13 mg/ml aprotinin (Sigma-Aldrich), and 2 mM PMSF (Santa Cruz Biotechnology). The suspended cells were sonicated for 10 s and then incubated on ice for 20 s. Sonication and cooling were repeated six times, and the cell lysate was centrifuged at 15,100*g* for 10 min to obtain cleared lysates. The total protein concentration in the lysates was determined using a Protein Assay Dye Reagent Concentrate. The crude lysates were stored at −80 °C until use.

The crude lysates were treated with calcium by mixing the crude lysates with the 5× reaction buffer (50 mM Tris-HCl [pH 7.6], 150 mM NaCl, 5 mM dithiothreitol, and 10 mM CaCl_2_) at a 4:1 ratio at 37 °C for 1 h. CaCl_2_ was omitted from the mock buffer that was used for experimental controls. To analyze phospho-STAT3, 10 mM sodium orthovanadate (Santa Cruz Biotechnology), a phosphatase inhibitor, was added to the reaction and mock buffers. The total protein content in the crude lysate for each reaction was 10 μg, unless otherwise stated. After incubation, the proteins in the crude lysates were analyzed by western blotting, as described above.

### Antibodies

Primary antibodies used for Western blot analysis are listed in Table 3. Antibodies were purchased from Abcam, Assay Genie (Dublin 1, Ireland), Bio-Rad, Cell Signaling Technology, Chemicon (Sigma-Aldrich), COSMO BIO, GeneTex, Invitrogen (Thermo Fisher Scientific), Medical Biological Laboratories, ProteinTech (Thermo Fisher Scientific), Sigma-Aldrich, and Thermo Fisher Scientific. Primary antibodies were detected using the following secondary reagents: horseradish peroxidase (HRP)-conjugated anti-rabbit IgG donkey polyclonal antibody (711-035-152, Jackson ImmunoResearch) and anti-rat IgG goat polyclonal antibody (112-035-167, Jackson ImmunoResearch) at a 1:40,000 dilution, and m-IgGκ BP-HRP (sc-516102, Santa Cruz Biotechnology) at a 1:10,000 or 30,000 dilution.

**Table 3:** Antibodies used in this study

Protein	References	Dilution for immunoblot
Actinin	Abcam, ab68167	1:5000
CAPN2	Cell Signaling Technology, 2539	1:000
CAPN2	Assay Genie, CAB4066	1:500
CAPN3	COSMO BIO, COP-080049	1:1000
CAPN5	GeneTex, GTX33057	1:3000
CAPN5	Bio-Rad, VPA00746	1:2000
GAPDH	Chemicon, MAB374	1:100,000–200000
HSP90	Santa Cruz, sc-13119	1:12,000
c-Myc,	Abcam, ab32072	1:4000
Myc tag	Invitrogen, R950-25	1:5000
MyoD	ProteinTech, 18943-1-AP	1:4000
Myogenin	Abcam, ab124800	1:2000
Myosin	Sigma-Aldrich, M4276	1:100
Phospho-STAT3	Cell Signaling Technology, 9145	1:2000
STAT3	Abcam, ab68153	1:2000
STAT5	Abcam, ab178941	1:5000
α-tubulin	Santa Cruz, sc-32293	1:1000–5000

### Giemsa staining

Myotubes grown in 60 mm culture plates (430166, Corning) in DM for 6 days were stained with May-Grünwald (63590, Sigma-Aldrich) and Giemsa solution (GS500, Sigma-Aldrich) according to a previously described method (30). Stained cells were visualized using an inverted microscope (CKX53, Olympus, Shinjuku, Tokyo, Japan) and LCAch N objective lenses at 10× magnification (0.25 NA), 20× magnification (0.40 NA), and 40× magnification (0.55 NA). Images were captured using a digital microscope color camera (DP23, Olympus).

### Immunocytochemistry

Cells were grown in 12-well culture plates and immunostained with an anti-myosin antibody (4276, Sigma-Aldrich; 1:400), biotin-conjugated anti-mouse IgG (BA-9200, Vector Laboratories), and Invitrogen streptavidin-conjugated Alexa Fluor 488 (S112223, Thermo Fisher Scientific), as previously described (47). Nuclei were counterstained with 5 μg/ml Hoechst 33342 (B2261, Sigma-Aldrich) in PBS for 10 min. Immunostained cells were observed under a microscope (IX-70, Olympus) using a Modulation Optics Hoffman modulation contrast apparatus (Navitar) and a 40× Plan-Semiapochromat objective lens (Modulation Optics HMC 40 LWD PL FL 0.6na∞/1, Navitar). Images were captured using an ORCA-ER-cooled charge-coupled camera (Hamamatsu Photonics) mounted on a microscope and processed using IPLab software (Scanalytics). The selected images were pseudo-colored for presentation using ImageJ.

## Data availability

All data are given in the manuscript.

## Supporting information

This article contains supporting information (24).

## Conflict of interest

The authors declare no conflicts of interest with the content of this article.

## Figures and Tables

**Fig. S4:**